# Editorial: Impact of microplastics on soil health and plant physiology in agricultural ecosystems

**DOI:** 10.3389/fpls.2025.1718582

**Published:** 2025-11-14

**Authors:** Surjit Singh, M. Naeem, Dominik K. Großkinsky, Ravikant Avasthe, Helena Freitas, Subhash Babu

**Affiliations:** 1 Department of Biotechnology, School of Health Sciences and Translational Research, Sister Nivedita University, New Town, West Bengal, India; 2 Department of Botany, Faculty of Life Sciences, Aligarh Muslim University, Aligarh, Uttar Pradesh, India; 3 AIT Austrian Institute of Technology, Center for Health & Bioresources, Bioresources Unit, Tulln an der Donau, Austria; 4 ICAR Research Complex for North Eastern Hill Region, Umiam, Sikkim, India; 5 Center for Functional Ecology, Department of Life Sciences, University of Coimbra, Coimbra, Portugal; 6 Division of Agronomy, ICAR-Indian Agricultural Research Institute, New Delhi, India

**Keywords:** agricultural sustainability, bioavailability, biodegradable plastics, heavy metal microplastics, soil–plant–microbe interactions

Wendell Berry quoted *“The soil is the great connector of our lives, the source and destination of all.”*, unfortunately, soil has recently emerged as the largest reservoir of microplastics (MPs), with concentrations estimated to be 4–23 times higher than those found in the oceans due to a variety of anthropogenic activities such as plastic mulching, sewage irrigation, unplanned municipal waste, littering, etc (Huang et al., 2021; Yang et al., 2021; Bian et al., 2022; Yu et al., 2022; Hoang et al., 2025). MPs are smaller particles (<5 mm) and are composed of different chemical components. They have emerged as a global environmental concern due to the extensive use and improper disposal of plastics. Their widespread occurrence has raised significant alarm over potential ecological risks and impacts on human health. In soils, microplastic pollution can profoundly influence key biogeochemical processes by altering soil properties, thereby affecting the activity and functional roles of soil microorganisms. These findings collectively point to the necessity of a precautionary approach in the use of plastics in agricultural systems, where even materials marketed as biodegradable may contribute to soil contamination and long-term ecosystem disruption.

The Asian region accounts for the most significant share of plastic production, producing 187.68 million tons of the global total, followed by Europe with 58.88 million tons. By 2050, plastic waste production is projected to reach 12,000 million tons (Ayeleru et al., 2020; Huang et al., 2023; Hoang et al., 2025). To date, the cumulative global production of plastics has already exceeded 8.3 billion tons (Plastics Europe, 2022). Despite this massive output, only around 20% of this plastic is recycled, while the remaining 80% accumulates in soils, rivers, and marine environments, contributing to widespread pollution (Plastics Europe, 2022; Azeem et al., 2023; Chaudhary et al., 2025; Hoang et al., 2025).

The agricultural landscape is undergoing unprecedented challenges as the invisible threat of MPs permeates soil ecosystems. Once hailed as revolutionary material, plastics now represent a looming ecological hazard, particularly in terrestrial environments that interact with crops, soil microbes, and co-pollutants such as heavy metals. The four recent studies reviewed here — investigating (i) microplastic-heavy metal interactions on lettuce and soil microbiota, (ii) metabolomic changes in highland barley under polystyrene microplastic exposure, (iii) the fate of biodegradable polylactic acid (PLA) MPs in maize, and (iv) soil quality and crop yield response to mulching strategies in organic eggplant production — collectively underscore the urgent need to rethink our approach to soil management and plastic use in agriculture.

## 1 MPs as emerging agroecosystem stressors

The study by Shirin et al. reveals the insidious risk posed by small-sized polystyrene MPs (13 µm), which exacerbate the toxicity of heavy metal-contaminated soils. Their work demonstrates how MPs serve as vectors, adsorbing cadmium, lead, copper, and arsenic, thereby increasing their bioavailability and uptake by lettuce. The consequence is a cascade of negative effects — suppressed plant growth, disrupted antioxidant systems, and altered soil microbial communities, with a noted decline in beneficial arbuscular mycorrhizal fungi and a rise in pathogenic fungi. Such shifts reveal a profound disturbance of rhizosphere ecology, where the delicate symbiosis between roots, mycorrhizae, and beneficial bacteria is destabilized, potentially impairing nutrient cycling and plant resilience across successive growing seasons. This highlights MPs as passive pollutants and active participants in shaping rhizosphere ecology.

## 2 Dose-dependent and biphasic plant responses

Interestingly, the toxicological narrative is not uniformly negative. Xiao et al. reported a concentration-dependent biphasic effect of PS-MPs on highland barley. At low (10 mg/L) and medium (50 mg/L) concentrations, biomass and root length significantly increased, suggesting a possible hormetic effect — where mild stress triggers adaptive metabolic responses. Metabolomic profiling indicated perturbation of flavonoid biosynthesis, purine/pyrimidine metabolism, and antioxidant pathways. At higher concentrations (100 mg/L), growth parameters declined sharply, reinforcing the threshold-dependent nature of microplastic phytotoxicity. These findings complicate the narrative and urge regulators to consider concentration-specific guidelines rather than binary classifications of safety versus hazard.

## 3 Biodegradable MPs: a solution or a new challenge?

Biodegradable plastics, particularly PLA, have been promoted as eco-friendly alternatives to conventional plastics. Bao et al. provide the first mechanistic evidence that PLA MPs not only undergo depolymerization in hydroponic solutions but also transform further within maize vascular systems. Low doses (1–10 mg/L) enhanced seed germination, shoot height, and biomass by over 50%, partly due to the release of dissolved organic carbon and stimulation of cell wall acidification via increased K^+^ flux. While these results suggest potential growth-promoting effects, they also raise important questions about the fate of degradation products and their accumulation in edible plant tissues. The risk of nanoparticle formation and translocation into the food chain cannot be overlooked, calling for long-term toxicological and nutritional studies before scaling up biodegradable plastic use in farming.

## 4 Mulching and soil health: the plastic dilemma

The benefits of plastic use in agriculture are undeniable, as highlighted by Amami et al. Black polyethylene mulch (BM) improved soil moisture retention, moderated temperature, and boosted eggplant yields by nearly 30 t/ha compared to bare soil. However, the breakdown of mulch into MPs must be weighed and compared to the short-term productivity gain, which contributes to the observed contaminated field soils worldwide. Moreover, the organic mulches (straw, compost) also promote microbial abundance and enhance the organic matter in the soil, offering a more sustainable path at par with the increased labour and management requirements.

## 5 Towards an integrated soil-plastic policy

These four studies converge on a crucial point, which states that an urgent need for a balanced, science-driven policy on agricultural plastic use is essential. The evidence suggests that MPs can cause stress and also stimulate plant growth, thus acting as both stressors and stimulators of plant physiological development. This mainly depends on size, concentration, and chemical nature of MPs. However, their potential to alter soil microbial community-driven network, mobilization of heavy metals to make bioavailable, and introduction of potential nanoplastic residues into edible crops requires an immediate precautionary approach. Therefore, an integrated soil management strategy must include:

### 5.1 Monitoring and regulation

Establishment of soil MP thresholds and regular checks on agricultural fields to monitor, particularly in high-risk areas such as industrial and mining-impacted regions.

### 5.2 Innovation in mulching

Invest in truly biodegradable mulch films that fully mineralize into CO_2_, water, and biomass without leaving persistent residues.

### 5.3 Microbiome-centered remediation

Explore the use of beneficial microbes and biochar to sequester MPs and immobilize heavy metals, restoring soil ecological balance.

### 5.4 Crop-specific risk assessment

Focused studies on leafy vegetables (e.g., lettuce, spinach, cabbage, etc.) and root crops should be prioritized that are directly prone to soil contaminants, as they are MPs’ key entry points into the food chain.

### 5.5 Circular economy approaches

Efforts in the reduction of plastic inputs at the source through the concept of reuse and recycling systems involved in agricultural practices, preventing MP accumulation for the future.

## Conclusion: from awareness to action

Microplastics are a rapidly growing concern which is challenging global food security, soil and environmental sustainability. The reviewed research provides valuable insights into how MPs (conventional or biodegradable) interact with crops, soil biogeochemistry, and microbial populations. It also underscores a paradox: plastics improve and deliver short-term agricultural productivity, but their long-term effect on soil resilience and food safety costs heavily on the ecological balance of the environment.

Today’s agricultural practices stand at a crossroads, where policymakers, researchers, and farmers must work together to redesign plastic use strategies that maximize the output of soil and crop management practices while reducing the ecological risks. Hence, a science-led policy framework is highly warranted to design plastic applications in agriculture. This must integrate systematic monitoring of microplastic residues in the soil-plant-human-environmental continuum, and the adoption of nature-based solutions for ecological balance. Additionally, crop and region-specific risk assessments and circular economy models will be central policy agenda for safeguarding soil and environmental resilience.

The series of MPs effects on the soil–plant–environment consortium ultimately affects the quality and safety of farm produce, raising legitimate concerns for both human and animal consumers. The subtle yet consistent accumulation of MPs and their associated contaminants in plant tissues underscores the urgency of adopting precautionary measures and designing new policies with immediate implementation scenarios to promote innovative remediation strategies. Hence, protecting soil health does not only focus on an ecological imperative but also on a cornerstone of sustainable food systems and public health protection.
